# MiR-3614-5p Is a Potential Novel Biomarker for Colorectal Cancer

**DOI:** 10.3389/fgene.2021.666833

**Published:** 2021-05-28

**Authors:** Lin Han, Yanjun Sun, Cansheng Lu, Chungeng Ma, Jian Shi, Dengqun Sun

**Affiliations:** ^1^Graduate School, Anhui University of Traditional Chinese Medicine, Hefei, China; ^2^Department of General Surgery, The Armed Police Corps Hospital of Anhui, Hefei, China; ^3^Department of Anus and Colon Surgery, The First Affiliated Hospital of Anhui University of Traditional Chinese Medicine, Hefei, China; ^4^Department of Anus and Colon Surgery, The Second Affiliated Hospital of Anhui University of Traditional Chinese Medicine, Hefei, China

**Keywords:** colorectal cancer, MiR-3614-5p, prognosis, TCGA, GSEA

## Abstract

MiR-3614-5p has been found in a variety of cancers including colorectal cancer. However, the association of miR-3614-5p with colorectal cancer is still unclear. Based on the Cancer Genome Atlas (TCGA) database, the relationship between miR-3614-5p and colorectal cancer can be proved. Wilcoxon rank-sum test was used to compare the miR-3614-5p expression in colorectal cancer tissues and under normal conditions, respectively. The logistic regression method was further employed to analyze the relationship between miR-3614-5p and clinicopathological characteristics. Also, the correlation between miR-3614-5p and survival rate was evaluated by Kaplan-Meier and Cox regression analysis. Besides, gene set enrichment analysis (GSEA) was used to investigate the biological functions of miR-3614-5p. The decrease of miR-3614-5p expression of colorectal cancer was significantly correlated with N stage (OR) = 0.7 for N1&N2 vs. N0), M stage (OR = 0.5 for M1 vs. M0), pathologic stage (OR = 0.7 for Stage III & Stage IV vs. Stage I & Stage II), neoplasm type (OR = 0.5 for rectum adenocarcinoma vs. colon adenocarcinoma), and lymphatic invasion (OR = 0.6 for YES vs. NO) (all *p*-values < 0.05). Kaplan-Meier survival analysis showed that colorectal cancer with low miR-3614-5p has a poorer prognosis than that of high miR-3614-5p (*p* = 0.005). According to univariate analysis, low miR-3614-5p was associated with poor overall survival (OS) [hazard ratio (HR) = 0.599; 95% confidence interval (CI): 0.418-0.857; *p* = 0.005]. In multivariate analysis, miR-3614-5p was closely related to OS (HR = 0.630; 95% CI: 0.405-0.978, *p* = 0.021). GSEA showed that the high expression phenotype of miR-3614-5p differentially enriches the P53 pathway. Meanwhile, the high expression phenotype of miR-3614-5p enhanced NK T cell activation, negative T cell selection, response to interleukin 2, and response to tumor cells. MiR-3614-5p is a possible prognostic marker of low survival rate for patients with colorectal cancer. Moreover, the P53 pathway and P38MAPK pathway may be the key pathways regulated by miR-3614-5p in colorectal cancer.

## Introduction

Colorectal cancer (CRC), a common malignant tumor in the gastrointestinal tract/intestine or large intestine, is a major threat to health around the globe. Nowadays, CRC is the fourth fatal cancer and causes nearly 900,000 deaths annually worldwide. In addition to the aging population and unhealthy eating habits in high-income countries, adverse risk factors such as the lack of physical exercise, obesity, and smoking also increase the risk of CRC (Dekker et al., 2019). CRC usually occurs when healthy colonic epithelial cells become benign adenomas and eventually end up in malignancies (Binefa et al., 2014). Through quantitative analysis of CRC estimates that mutation from stem cells to malignant cells happens at first. Then it takes some time for these tumor cells to acquire metastatic ability. The window period is about 10 years (Jones et al., 2008). Therefore, early diagnosis of CRC is a challenging task for clinicians. According to statistics of CRC patients, nearly 43% of patients have liver metastases and 25% of patients have liver and lung metastases. Besides, the 5-year survival rate of stage IV is less than 10% (Chuang et al., 2017; Bora et al., 2021). Surgery remains to be the primary clinical treatment, supplemented by chemotherapy and immunotherapy. However, the efficacy of these treatments remains poor for the advanced stage of the disease. In summary, there is great urgency to develop new diagnostic and therapeutic targets.

MicroRNAs (miRNAs) are small non-coding RNAs with a size of 19-25 nucleotides (Bartel, 2004; Chen et al., 2019). Due to their short structure, they may be crucial biomarkers. They are easy to be used in the early detection and treatment of various cancers and have been proved workable in real practice (Kapp et al., 2015). Moreover, miRNA is of great use in regulating biological and pathological processes throughout the development of cancer (Bartel, 2004; Lin and Gregory, 2015).

Exosomes derived from tumor cells have an active role in carcinogenesis, metastasis, and response to treatment through the transfer of oncogenes and onco-miRNAs between CRS and tumor stroma cells (Nedaeinia et al., 2017). MiRNA, which is often dysregulated in cancer, has shown great potential as a tissue-based marker for cancer classification and prognosis (Hayes et al., 2014; Okugawa et al., 2015). Certain miRNAs could be secreted into the blood as cell-free miRNAs. They can be detected in serum as a highly stable form. Therefore, circulating miRNA has become a promising invasive biomarker for the diagnosis and monitoring of human cancers (Zen and Zhang, 2012; Huang et al., 2017).

MicroRNAs can act as biomarkers in colorectal cancer (Schee et al., 2010).

MiR-3614-5p with a length of 24 nt was located on chromosome 17q22. It has been reported that miR-3614-5p can antagonize the dengue virus by regulating adenosine deaminase which acts on RNA 1 (ADAR1) in human macrophages (Diosa-Toro et al., 2017). It is reported that miR-3614-5p plays a crucial role in the progress of non-small cell lung cancer. These findings may provide potential targets for the development of treatment strategies for patients with non-small cell lung cancer (Li et al., 2020). Besides, overexpression of miR-3614-5p significantly inhibited the proliferation of breast cancer cells (Wang et al., 2019). Bioinformatics analysis showed that miR-3614-5p may inhibit the WNT signaling pathway by targeting NFATC2 in NSCLC cells (Shang et al., 2019). Another report shows that there is a close relationship between the expression of miR-3614-5p and the risk of autoimmune diseases (Wohlers et al., 2018). Previous studies also used a miRNA risk-stratification signature that can be used as a non-invasive assay for the identification of high-risk patients and potential disease monitoring in patients with PDAC (Kandimalla et al., 2020; Lv et al., 2020). MicroRNAs are involved in many biological and pathological processes such as cell growth, differentiation, apoptosis, etc. Dysregulation of miRNAs expression patterns has been reported in many tumors including Colorectal Cancer. Various studies indicate that miRNAs can be utilized as diagnostic and prognostic biomarkers for evaluation of tumor initiation, development, invasion, metastasis, and response to chemotherapeutic drugs. Numerous investigations have also shown dysregulation of miRNAs in tissue samples and body fluids such as serum, plasma, and fecal samples from CRC patients (Shirafkan et al., 2018).

In this study, CRC-related miRNA-Seq data were acquired from the Cancer Genome Atlas (TCGA) for further accessing the prognostic value of the miR-3614-5p expression. It was found that CRC patients with miR-3614-5p have significantly lower expression. Thus, the miR-3614-5p can be a sensitive biomarker and prognosis standard.

## Materials and Methods

### Sequencing Data and Clinic Information of miRNA From TCGA Data Repository

The level 3 BCGSC miRNA profiling miRNA-Seq data and clinical information were downloaded from the TCGA^1^ colorectal cancer COAD and READ projects. Among the statistics, miRNA-Seq data without clinical information were discarded. A total of 602 miRNA-Seq data with clinical information were used for further association analysis (Table 1). The detailed clinicopathologic characteristics including age, pathologic stage, neoplasm type (rectum adenocarcinoma vs. colon adenocarcinoma), height, weight, gender, race, history of colon polyps, colon polyps present, and lymphatic invasion were recorded for analysis. Besides, other information for records included TP53 status, KRAS status, PIK3CA status, and TNM stage (TNM stage is the tumor stage, where T refers to the tumor, representing the range of primary tumor; N is a lymph node, showing whether there are lymph node metastasis and the range of the representative area; and M represents the existence of a distant transfer). This study does not include direct research of human participants or animals exerted by any authors.

**TABLE 1:** TCGA colorectal cancer patient characteristics.

Clinical characteristics	Level	Overall (602)
T stage (%)	T1	20 (3.3%)
	T2	101 (16.8%)
	T3	411 (68.5%)
	T4	68 (11.3%)
N stage (%)	N0	337 (56.3%)
	N1	148 (24.7%)
	N2	114 (19.0%)
M stage (%)	M0	441 (83.4%)
	M1	88 (16.6%)
Pathologic stage (%)	Stage I	102 (17.5%)
	Stage II	216 (37.1%)
	Stage III	175 (30.1%)
	Stage IV	89 (15.3%)
Gender (%)	Female	285 (47.3%)
	Male	317 (52.7%)
Lymphatic invasion (%)	NO	322 (59.5%)
	YES	219 (40.5%)
History of colon polyps (%)	NO	354 (68.9%)
	YES	160 (31.1%)
Colon polyps present (%)	NO	208 (69.3%)
	YES	92 (30.7%)
Neoplasm type (%)	Colon adenocarcinoma	442 (73.4%)
	Rectum adenocarcinoma	160 (26.6%)
TP53 status (%)	Mut	306 (59.3%)
	WT	210 (40.7%)
KRAS status (%)	Mut	207 (40.1%)
	WT	309 (59.9%)
PIK3CA status (%)	Mut	130 (25.2%)
	WT	386 (74.8%)
Age (median [IQR])		68.00 (58.00, 76.00)
Height (median [IQR])		170.00 (162.00, 176.00)
Weight (median [IQR])		78.90 (65.00, 92.00)

**IQR, InterQuartile Range; WT, wild type; MUT, mutant.**

### Gene Set Enrichment Analysis

Gene Set Enrichment Analysis (GSEA) is a computational method and determines whether predetermined gene sets have statistically significant and consistent differences between two biological states (Subramanian et al., 2005). Based on the co-expression gene analysis of miR-3614-5p, GSEA was performed on the miR-3614-5p low and high expression groups through the TCGA colorectal cancer COAD and READ expression matrix, and clusterPorfiler package (Yu et al., 2012). In this study, an ordered list of all genes was made based on their correlation with miR-3614-5p expression via GSEA. Then GSEA was performed to clarify the significant observed survival differences between the high and low miR-3614-5p groups. We used a preliminary version of GSEA to analyze data (Mootha et al., 2003). Each analysis performs 10,000 permutations of the genome. The level of miR-3614-5p expression = functions as a phenotypic marker. By adjusting the *p*-value, the enrichment pathway for each phenotype was classified via the standardized enrichment score (NES).

### Analysis of Immune Cell Characteristics by ssGSEA

The CRC immune infiltrate was analyzed by ssGSEA (single sample GSEA GSEA) and GSVA package in R (3.6.3) (Barbie et al., 2009). Thereby, GSEA was conducted on 24 types of immune cells in tumor samples, which include B cells, CD8 T cells, Eosinophils, Mast cells, Treg, NK CD56 bright cells, Th1 cells, immature DCs[iDCs], DCs, Th17 cells, T helper cells, NK cells, T cells, NK CD56 dim cells, Tgd, pDCs, Tfh, T effector memory[Tem], Cytotoxic cells, Tcm, Neutrophils, activated DCs[aDCs], Macrophages and Th2 cells. Then, according to the characteristic genes of 24 immune cells in the literature (Bindea et al., 2013), the relative enrichment fraction of each immune cell was quantified from the gene expression profile of each tumor sample. Spearman correlation and Wilcoxon rank-sum test analysis were used for analyzing the correlation between miR-3614-5p and immune cell infiltration level and the correlation between immune cell infiltration and different expression groups of miR-3614-5p.

### Statistical Analysis

All statistical analyses were conducted using R (3.6.3). Wilcoxon rank sum test and logistic regression were used for analyzing the relationship between clinicopathological characteristics and miR-3614-5p. Cox regression and Kaplan-Meier method were employed in the analysis of clinical and pathological characteristics related to overall survival (OS) in TCGA patients. Multivariate Cox analysis was used to compare the effect of the miR-3614-5p expression on survival and other clinical characteristics (pathological stage, tumor type, gender, history of colon polyps, presence of colon polyps, lymphatic invasion, TP53 status, KRAS status, and PIK3CA status). The critical value of the miR-3614-5p expression was determined by its median value (in all tests, *p*-values less than 0.5 were considered significant).

## Results

### Patients and Samples

As shown in Table 1, the characteristics of patients may affect survival rate. A total of 285 female patients and 317 male patients were analyzed in this study. Among them, patients with lymphatic invasion account for 219 (40.5%). 31.1% (*n* = 160) had a history of colon polyps before treatment, and 92 cases (30.7%) had colonic polyps. Following the statistical analysis of the pathologic stage, patients with stage I accounted for 17.5% (*n* = 102), while 37.1% (*n* = 216), 30.1% (*n* = 175), and 15.3% (*n* = 89) patients were in stage II, III, and IV, respectively. In the light of the anatomy, 442 tumors cases (73.4%) were colon adenocarcinoma, and 160 cases (26.6%) were rectal adenocarcinoma. Meanwhile, the topography distribution contained T stage: 3.293% T1 (*n* = 20), 16.8% T2 (*n* = 101), 68.5% T3 (*n* = 411), and 11.3% T4 (*n* = 68); N stage: 56.3% N0 (*n* = 337), 24.7% N1 (*n* = 148), 19.0% N2 (*n* = 114); and M stage: 83.4% M0 (*n* = 441), 16.6% M1 (*n* = 88). Besides, the expression of TP53, KRAS and PIK3CA status were, respectively, studied according to wild type (WT) and mutant (MUT).

### The Expression and Diagnostic Value of miR-3614-5p in Colorectal Tissues

**FIGURE 1:**
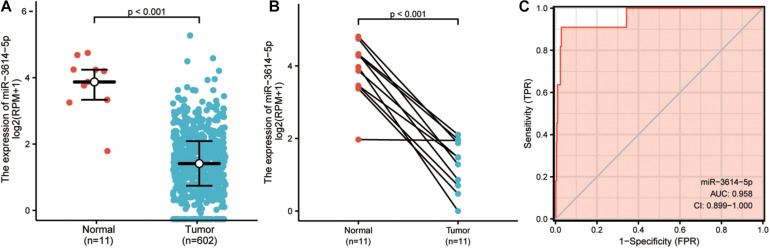
The expression and diagnostic value of miR-3614-5p in colorectal tissues. **(A)** miR-3614-5p showed significantly lower expression in cancer tissues than in normal tissues. **(B)** miR-3614-5p was prominently lowexpressed in colorectal cancer (*P* < 0.001) compared with 11 pairs non-cancerous adjacent tissues. **(C)** Receiver Operating Characteristic Curve, FPR, False Positive Rate; TPR, True Positive Rate; CI, Confidence interval.

Next, Wilcoxon rank-sum test was used to examine the expression of miR-3614-5p in 602 CRC tissues and 11 normal tissues. The expression of miR-3614-5p in cancer tissues was much lower than that in normal ones (*P* < 0.001) (Figure 1A). Besides, the Wilcoxon single-rank test was employed to further analyze the expression of miR-3614-5p in 11 pairs of CRC tissues and healthy adjacent tissues. The result shows that miR-3614-5p was prominently low-expressed in CRC (*P* < 0.001) (Figure 1B), indicating that miR-3614-5p may facilitate the occurrence of CRC. From the TCGA database, ROC (receiver operating characteristics) was used for predicting the outcome of CRC and adjacent tissues. The diagnostic efficacy of miR-3614-5p for CRC was also analyzed. The area under the curve (AUC) of miR-3614-5p in Figure 1C is 0.958, which indicates that the expression of miR-3614-5p has a good discrimination ability in tumor and healthy tissues (Under the ROC curve area value from 0.5 to 1, the closer AUC is to 1, the better the diagnostic effect will be. AUC 0.5 ∼ 0.7 leads to lower accuracy, while AUC 0.7 ∼ 0.9 results in moderate accuracy, and higher accuracy can be guaranteed as AUC is higher than 0.9).

### Association Between miR-3614-5p Expression Level and Clinicopathologic Variables

**FIGURE 2:**
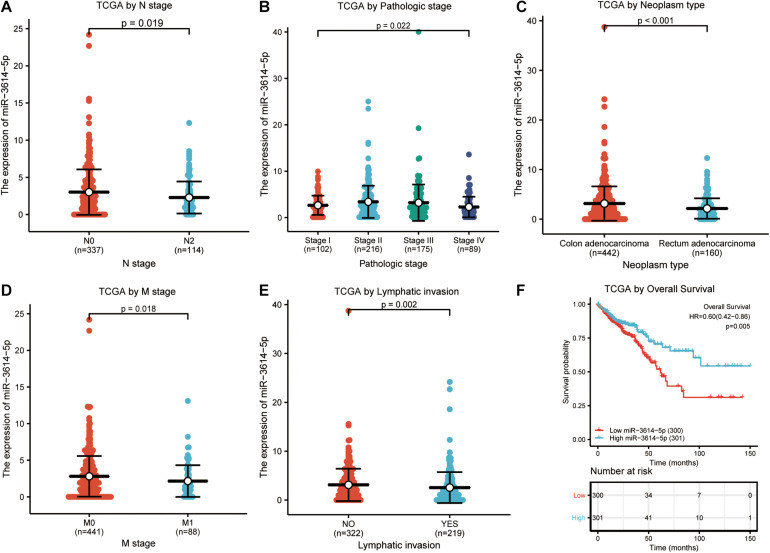
Correlation analysis between miR-3614-5p expression and clinicopathological factors. **(A)** N stage. **(B)** Pathologic stage. **(C)** Neoplasm type. **(D)** M stage. **(E)** Lymphatic invasion. **(F)** Effect of miR-3614-5p expression on OS of CRC patients in TCGA cohort. TCGA, The Cancer Genome Atlas; OS, overall survival; CRC, colorectal cancer.

In total, 602 CRC samples with miR-3614-5p expression data were analyzed from TCGA, which covered all characteristics of the patients. As shown in Figures 2A–E, the low expression of miR-3614-5p was significantly correlated with tumor N stage (N2 vs. N0, *p* = 0.019), pathological stage (Stage I vs. Stage II vs. Stage III vs. Stage IV, *p* = 0.022), tumor type (Rectum adenocarcinoma vs. colon adenocarcinoma, *p* < 0.001), M stage (M1 vs. M0, *p* = 0.018), and lymphatic invasion (YES vs. NO, *p* = 0.002). The results of the univariate analysis which employs logistic regression show that miR-3614-5p expression as a categorical dependent variable (based on median expression value of 2.5) is related to poor prognostic clinicopathological characteristics (Table 2). Decreased expression of miR-3614-5p in CRC was related to the following factors: N stage (OR of N1 and N2 and N0 = 0.7), M stage (OR of M1 and M0 = 0.5), pathological stage (OR of stage III and IV = 0.7 and I Stage and II), tumor type (OR of rectal adenocarcinoma and colon adenocarcinoma = 0.5), and lymphatic infiltration (OR of YES vs. NO = 0.6) (all *p* < 0.05). These results indicate that compared with people with high miR-3614-5p expression, people with low miR-3614-5p expression are more likely to enter the late stage and may have a lymphatic invasion.

**TABLE 2:** miR-3614-5p expression* associated with clinical pathological characteristics (logistic regression).

Characteristics	Total (N)	Odds Ratio in miR-3614-5p expression	*P*-value
T stage (T3&T4 vs. T1&T2)	600	0.87 (0.58–1.30)	0.502
N stage (N1&N2 vs. N0)	599	0.69 (0.50–0.95)	0.023
M stage (M1 vs. M0)	529	0.52 (0.32–0.83)	0.007
Pathologic stage (Stage III&Stage IV vs. Stage I&Stage II)	582	0.68 (0.49–0.95)	0.023
Neoplasm type (Rectum adenocarcinoma vs. Colon adenocarcinoma)	602	0.50 (0.34–0.72)	<0.001
Lymphatic invasion (YES vs. NO)	541	0.62 (0.44–0.88)	0.007
TP53 status (Mut vs. WT)	516	1.08 (0.76–1.54)	0.652
KRAS status (Mut vs. WT)	516	0.94 (0.66–1.34)	0.730
PIK3CA status (Mut vs. WT)	516	1.04 (0.70–1.55)	0.844

*C*ategorical dependent variable, greater or less than the median expression level.*

### Univariate and Multivariate Survival Analyses

According to Figure 2F, Kaplan-Meier survival analysis shows that CRC with low miR-3614-5p was associated with a worse prognosis than CRC with high miR-3614-5p (*p* = 0.005). Univariate analysis shows that low miR-3614-5p was closely related to worse OS [hazard ratio (HR): 0.599; 95% confidence interval (CI): 0.418–0.857; *p* = 0.005]. Other clinicopathological variables related to low survival rate include TNM stage, pathological stage, age, and lymphatic invasion. In multivariate analysis, miR-3614-5p was independently associated with OS, HR of 0.630 (95% CI: 0.405–0.978, *p* = 0.021), as well as M stage, pathological stage, and age (Table 3).

**TABLE 3:** Univariate and multivariate Cox proportional hazards regression analysis of miR-3614-5p expression.

Characteristics	Total (N)	HR (95% CI) univariate analysis	*P-*value univariate analysis	HR (95% CI) multivariate analysis	*P*-value multivariate analysis
T stage (T3&T4 vs. T1&T2)	599	2.383 (1.281–4.434)	0.006		
N stage (N1&N2 vs. N0)	598	2.630 (1.826–3.788)	<0.001		
Lymphatic invasion (YES vs. NO)	540	2.076 (1.422–3.032)	<0.001		
M stage (M1 vs. M0)	528	4.087 (2.741–6.092)	<0.001	2.126 (1.256–3.599)	0.005
Pathologic stage (Stage III&Stage IV vs. Stage I&Stage II)	581	2.999 (2.040–4.409)	<0.001	6.678 (1.985–22.461)	0.002
Age (> 65 vs. == 65)	601	2.034 (1.377–3.005)	<0.001	3.088 (1.908–4.997)	<0.001
miR-3614-5p (High vs. Low)	601	0.599 (0.418–0.857)	0.005	0.630 (0.405–0.978)	0.039

**HR, hazard ratio.**

### GSEA Identifies a miR-3614-5p-Related Signaling Pathway

**FIGURE 3:**
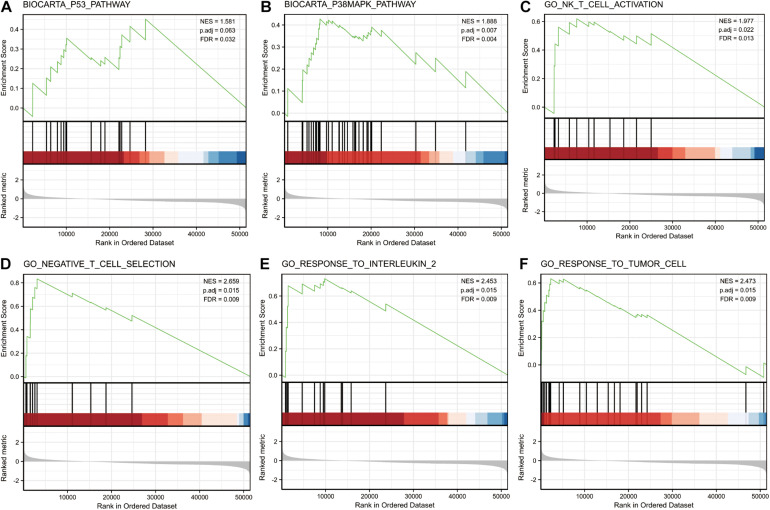
Enrichment plots from gene set enrichment analysis (GSEA). GSEA results showing P53 pathway **(A)**, P38MAPK pathway **(B)**, NK T cell activation **(C)**, negative T cell selection **(D)**, response to interleukin 2 **(E)** and response to tumor cell **(F)** are differentially enriched in miR-3614-5p-related CRC. NES, normalized ES; p.adj, adjust *p-*value; FDR, false discovery rate.

GSEA was performed on the low and high miR-3614-5p expression datasets to identify signaling pathways that are differentially activated in CRC. It revealed significant differences in the enrichment of MSigDB collections (c2.cp.v7.0. and c5.all.v7.0. symbols) (FDR < 0.05, NOM *p*-val < 0.05). The most enriched signaling pathways were selected based on the normalized enrichment score (NES) (Figure 3 and Table 4). Figures 3A–F illustrates that the high expression phenotype of miR-3614-5p differentially enriches the P53 pathway, P38MAPK pathway, NK T cell activation, negative T cell selection, response to interleukin 2, and response to tumor cells.

**TABLE 4:** Gene sets enriched in phenotype high.

MSigDB collection	Gene set name	NES	p.adj	FDR
c2.cp.v7.0. symbols.gmt	BIOCARTA_P53_PATHWAY	1.581	0.063	0.032
	BIOCARTA_P38MAPK_PATHWAY	1.888	0.007	0.004
c5.all.v7.0. symbols.gmt	GO_NK_T_CELL_ACTIVATION	1.977	0.022	0.013
	GO_NEGATIVE_T_CELL_SELECTION	2.659	0.015	0.009
	GO_RESPONSE_TO_INTERLEUKIN_2	2.453	0.015	0.009
	GO_RESPONSE_TO_TUMOR_CELL	2.473	0.015	0.009

**NES, normalized enrichment score; p.adj, adjust p-value; FDR, false discovery rate.**

**TABLE 4-1:** The corresponding transcript names for each pathway in this table.

Gene set name	Corresponding transcript name
BIOCARTA_P53_PATHWAY	BCL2/MDM2/ATM/CDK2/APAF1/CCND1/RB1/CCNE1/CDKN1A/E2F1/PCNA/BAX/CDK4/GADD45A/TP53/TIMP3
BIOCARTA_P38MAPK_PATHWAY	STAT1/RPS6KA5/MAP3K5/PLA2G4A/CDC42/GRB2/MAX/HMGN1/MAP3K1/CREB1/MAP2K6/DDIT3/MAP2K4/ATF2
GO_NK_T_CELL_ACTIVATION	IL6R/IL12B/IL18/IL15/RASAL3/CD300A
GO_NEGATIVE_T_CELL_SELECTION	PTPRC/THEMIS/DOCK2/CD74/CCR7/CD3E/CD28
GO_RESPONSE_TO_INTERLEUKIN_2	CITED1/IL2/IL2RB/IL2RA/JAK3/PTPN2/JAK1/STAT5A/CDC5L
GO_RESPONSE_TO_TUMOR_CELL	CD274/CRTAM/KLRC4-KLRK1/CD226/KLRK1/PRF1/HAVCR2/CD160/IL12B

### The Correlation Between miR-3614-5p Expression and Immune Infiltration

**FIGURE 4:**
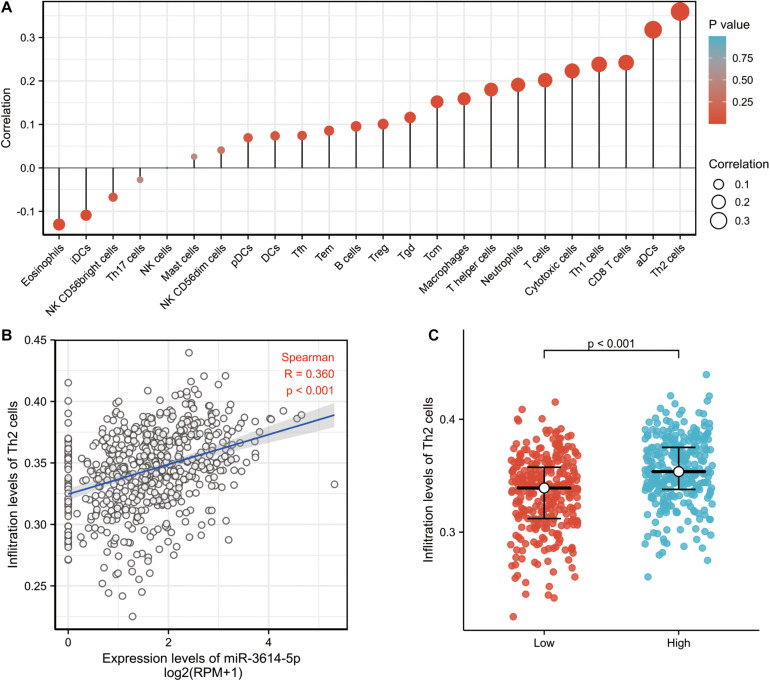
The correlation between miR-3614-5p expression and immune invitation. **(A)** The forest plot shows the correlation between miR-3614-5p expression and immune cell subsets. **(B)** Correlation between the relative enrichment score of Th2 cells and the expression level (TPM) of miR-3614-5p. **(C)** Difference in Th2 cells infiltration level between miR-3614-5p high and low expression groups.

Then, the correlation between the expression level (TPM) of miR-3614-5p and immune cell enrichment level (generated by ssGSEA) was analyzed by Spearman correlation. As a result, the miR-3614-5p expression was found negatively correlated with the abundances of immunocytes (Eosinophils, iDCs, NK CD56bright cells, etc.), while positively correlated with the abundances of immunocytes (Th2 cells, aDCs, CD8 T cells, Th1 cells, Cytotoxic cells, T cells, etc.) (Figure 4A). Wilcoxon rank sum test also showed that the enrichment score of Th2 cells was significantly higher in miR-3614-5p high expression samples (Figure 4B). Furthermore, the difference in Th2 cells infiltration level was analyzed in miR-3614-5p high and low expression groups. The results are statistically significant (*p* < 0.001) (Figure 4C).

### Compute the Matthews Correlation Coefficient (MCC)

Simultaneously, we recalculate the matthews correlation coefficient (MCC) value by querying the data on colorectal cancer in TCGA (Bandyopadhyay et al., 2014). Use the calculation formula of MCC to substitute the value into the formula. After the calculation, the MCC value is 0, it can be understood as an average random prediction, which can better reflect a correct prediction in this study (the MCC is in essence a correlation coefficient between the observed and predicted binary classifications; it returns a value between -1 and + 1. A coefficient of + 1 represents a perfect prediction, 0 an average random prediction and -1 an inverse prediction).

## Discussion

According to recent studies, miR-3614-5p plays an essential role in various cancers. For example, it can be concluded that overexpression of CHAIP2 may hinder LUAD cell proliferation and invasion due to the modulation of the WNT signal pathway targeted by miR-3614-5p (Shang et al., 2019). The genome-wide association study (GWAS) signal for rheumatoid arthritis and Crohn’s disease were enriched in genes predicted to be targeted by miR-3614-5p. What’s more, it points out the potential pathophysiological role of miR-3614-5p in autoimmunity (Wohlers et al., 2018). However, few studies have been carried out on the correlation between miR-3614-5p and CRC. However, little correlation has been proved between miR-3614-5p and CRC. Therefore, this paper is aimed at clarifying the expression of miR-3614-5p in CRC tissue, and its potential therapeutic and prognostic value. In this research, the miR-3614-5p was significantly downregulated in CRC tissue compared to normal or adjacent normal tissue. Thus, the potential role of miR-3614-5p low expression in CRC is the focus of the present study.

Herein, CRC data based on high throughput RNA sequencing were collected from the TCGA database. Meanwhile, it was demonstrated that miR-3614-5p was significantly down-regulated in CRC tissues compared with normal or adjacent normal tissues. It is proved that reduced expression of miR-3614-5p in CRC is associated with clinical pathologic characteristics in advanced periods (age, clinical stage, pathologic stage, histological type, lymphatic invasion), short survival time, and poor prognosis. Furthermore, GSEA results show that miR-3614-5p phenotypes with high expression are significantly related to the P53 pathway, P38MAPK pathway, NK T cell activation, negative T cell selection, response to interleukin 2, and response to the tumor cell. These pathways are said to be responsible for the proliferation of cancer cells, invasion, and metastasis (Fan et al., 2014; Wang et al., 2017; Kimura, 2018; Yu-Lee et al., 2018; Najafi et al., 2019). The results suggest that the miR-3614-5p may be a new therapeutic and prognostic target for CRC. Studies have shown that Brahma-related gene 1 (BRG1) plays an important role in cell aging and tumor growth. Thus, it promotes a new mechanism of cell senescence in CRC by affecting the p53 signal axis, which is a new potential target for cancer treatment. Current studies have shown that there are functionally important single nucleotide polymorphisms (SNPs) in certain genes of the pathway p53. They can change the signal transduction amplitude of the protein. Besides, those variants may influence cancer risk, progression, and efficacy of radiation and chemotherapy. Besides, the p53 pathway is of great significance in other biological processes, including metabolism and reproductive adaptability. Thereby, these variants also have the potential to change other diseases (Basu and Murphy, 2016). Studies have also shown that the migration and invasion of human gastric cancer cells SGC7901 can be inhibited by the P38MAPK signaling pathway through the expression of MMP-2 and MMP-9 (Lu et al., 2017). This study further tests the phosphorylation of P38MAPK to completely stratify the prognosis, showing the combined survival advantage of P38MAPK in cMMR BRAF mutant stage III CRC patients (Roseweir et al., 2018). Studies have shown that Barbaloin increases the apoptotic rate of A549 cells by reducing cell growth and Ki-67 expression levels while proliferates cell nuclear antigen (PCNA).

Besides, Barbaloin induces G2/M phase accumulation, thereby inactivating the P38MAPK signaling pathway. In the end, the proliferation and metastasis of small cell lung cancer can be inhibited (Zhang et al., 2017). Phosphorylated p38 (p-p38) is important in the regulation of disease progression. It is related to tumor prognosis. Poor OS of CRC can be predicted by overexpression of p-p38, which is also an important independent factor leading to death, recurrence, and distant metastasis (Fan et al., 2014). Studies have shown that the overexpression of methyltransferase 3 (METTL3) exerts a tumor suppressor effect on the proliferation, migration, and invasion of CRC cells through the p38 pathway (Deng et al., 2019). This indicates that miR-3614-5p can affect the growth, metastasis, and low survival rate of colorectal cancer cells through the P53 and P38MAPK pathways.

Another important aspect of this study is that the expression of miR-3614-5p is correlated with multiple levels of immune infiltration in CRC. What’s more, the results also indicate that in CRC expression, there is a moderate to a strong positive correlation between the expression level of miR-3614-5p and the infiltration level of Th2 cells and aDC. Besides, the infiltration level of CD8 T cells, Th1 cells, cytotoxic cells, and T cells have significant positive correlations with the expression of miR-3614-5p. Besides, the correlation between miR-3614-5p expression and immune cell marker genes suggests the role of miR-3614-5p in regulating CRC tumor immunology. First, iDC has a weak correlation with miR-3614-5p expression, while aDC shows a strong correlation. These results reveal the potential regulatory role of miR-3614-5p in tumor-related DC polarization. In addition, a significant correlation can be found between the expression of miR-3614-5p and the regulation of several markers of T helper cells in CRC (Th2, Th1, Tfh). These correlations may indicate the potential mechanism by which miR-3614-5p regulates T cell function in CRC. These findings collectively indicate that miR-3614-5p may largely affect the recruiting and regulating of immune infiltrating cells in CRC.

In this paper, these methods deepened our understanding of the relationship between miR-3614-5p and CRC. However, further improvements need to be made. First, to fully reveal the specific role of miR-3614-5p in the occurrence of CRC, various clinical factors, such as detailed information about the patients being treated should be considered. However, considering that the experiments are conducted in multiple laboratories, the processing methods in the public database may be inconsistent. Second, the number of healthy subjects used for controls in this study is significantly variant from the number of cancer patients. As a consequence, follow-up studies are needed to maintain a balanced sample size. Finally, multi-center studies in public databases are designed to make up for the shortcomings of single-center studies. However, there are also limitations in retrospective studies, especially differences in interventions and the lack of specific information. As a result, prospective studies are supposed to be conducted in the future to avoid analysis bias due to the retrospective nature of the current research. Moreover, it is impossible to clearly assess the direct mechanism of miR-3614-5p involved in CRC development. Therefore, future research should carry out wet experiments on the direct mechanism of CRC.

## Conclusion

According to our study, low miR-3614-5p expression is closely associated with CRC cancer progression, low survival rate, and immune infiltration. Thus, it can promote tumor formation through abnormal inflammation and immune responses. This study provides promising insights to exhume the clinical-pathological significance and molecular etiology of CRC. However, further randomized clinical trials and supplementary studies are needed to validate the basic molecular mechanism and clinical application of CRC patients.

## Data Availability Statement

The datasets presented in this study can be found in online repositories. The names of the repository/repositories and accession number(s) can be found in the article/supplementary material.

## Author Contributions

DS: conceptualization and design of the study and funding. LH, YS, and CL: details of the experimental design. LH: carrying out the experiment, preparation of the data. CM, JS, YS, and LH: implementation of the scenario. DS and LH: analysis. LH, DS, and YS: writing of the manuscript. All authors contributed to the article and approved the submitted version.

## Conflict of Interest

The authors declare that the research was conducted in the absence of any commercial or financial relationships that could be construed as a potential conflict of interest.

## Footnotes

1 https://portal.gdc.cancer.gov/
